# Crosstalk between Exercise and Immunotherapy: Current Understanding and Future Directions

**DOI:** 10.34133/research.0360

**Published:** 2024-04-25

**Authors:** Jiwei Liu, Weici Liu, Yuan Wan, Wenjun Mao

**Affiliations:** ^1^ Department of Thoracic Surgery, The Affiliated Wuxi People’s Hospital of Nanjing Medical University, Wuxi People’s Hospital, Wuxi Medical Center, Nanjing Medical University, Wuxi 214023, China.; ^2^ Wuxi College of Clinical Medicine, Nanjing Medical University, Wuxi 214023, China.; ^3^ The Pq Laboratory of BiomeDx/Rx, Department of Biomedical Engineering, Binghamton University, Binghamton, NY 13850, USA.

## Abstract

Accumulated evidence highlights that exercise can modulate multiple cytokines, influencing transcriptional pathways, and reprogramming certain metabolic processes, ultimately promoting antitumor immunity and enhancing the efficacy of immune checkpoint inhibitors in cancer patients. Exploring the mechanisms behind this will, for one thing, help us uncover key factors and pathways in exercise-assisted cancer immunotherapy, offering more possibilities for future treatment methods. For another, it will support the development of more personalized and effective exercise prescriptions, thereby improving the prognosis of cancer patients.

## Introduction

The advent of immunotherapy has brought about a transformative shift in cancer treatment, transcending the traditional focus on solely targeting tumor cells, instead embracing the crucial role of the tumor immune microenvironment. However, due to the intricate nature of the immune system and the diverse characteristics of tumors, the response to immunotherapy can vary significantly, resulting in limited benefits [1]. Moreover, the clinical application of immunotherapy efficacy-predictive biomarkers, such as tumor mutational burden and single-gene mutations, is also very limited [2].

The composition and functional states of immune cells within the tumor microenvironment (TME) are crucial for the response to immunotherapy [1]. The degree of immune cell infiltration and the levels of inflammatory factors serve as indicators of the intensity of immune response, allowing for the classification of the TME into either “immune-cold” or “immune-hot” types. In general, immune-hot tumors are characterized by an abundance of infiltrating immune cells and heightened immune activity, contributing to a more robust response.

Evidence demonstrates that exercise reduces the risk of morbidity and all-cause mortality in a wide range of cancers [3]. In addition, a compelling association exists between exercise and optimistic prognoses for cancer patients, for that exercise has the potential to improve the quality of life for cancer patients. This is thought to be in correlation with enhanced anticancer immune responses, which can ultimately improve the effectiveness of immunotherapy [4].

## Exercise Augments the Efficacy of Immunotherapy

The engagement in physical exercise can promote the mobilization of specific immune cell subsets and potentiate their cytotoxicity, differentiation, and migration [4], indicating that physical exercise can improve the TME, paving the way for the enhanced efficacy of immunotherapy. Notably, the synergistic effects between exercise and immunotherapy have been proved in multiple preclinical tumor models. Martín-Ruiz et al. [5] reported a remarkably higher tumor necrosis in mice with lung cancer receiving exercise and immune checkpoint inhibitors (ICIs). Similarly, in combination with programmed cell death protein 1 (PD-1) antibody therapy in melanoma mice, exercise considerably reduced tumor weight and volume, demonstrating a more potent tumor growth inhibition. Histological staining further corroborated the remarkable synergistic effect of the combination treatment, revealing a greater extent of tumor cell apoptosis and proliferation inhibition. To our greater satisfaction, no exercise-related adverse events were observed, supporting the safety and feasibility of the joint strategy to enhance the efficacy [6].

What is noteworthy is that exercise also potentiates the effectiveness of the dual antitumor therapy, which consists of immunotherapy and other therapies. For example, exercise markedly boosted the responses of breast cancer mice to focal radiotherapy and PD-1 blockade, resulting in a significantly slower rate of tumor growth [7]. Similar benefits were also shown in hepatocellular carcinoma patients engaged in exercise alongside lenvatinib and anti-PD-1 therapy, whose 1- and 2-year overall survival rates were 92.7% and 77.3%, respectively, compared to 67.1% and 58.7% in the sedentary group. Notably, their median progression-free survival (PFS) was 17 months longer than that of the nonexercisers. So was the objective response rate (ORR), with 57.1% in the exercise group vs 22.6% in the sedentary group. This implies that patients engaging in exercise have lower risks of death and progression, as well as a higher likelihood of achieving objective responses. Subsequent animal experiments further confirmed that exercise remarkably suppressed the infiltration of regulatory T cells and the expression of immune checkpoints, leading to an extended survival and an improved efficacy [8].

## Mechanistic Insights into Exercise-Enhanced ICI Efficacy

### Regulating multiple cytokines

**Fig. 1.**
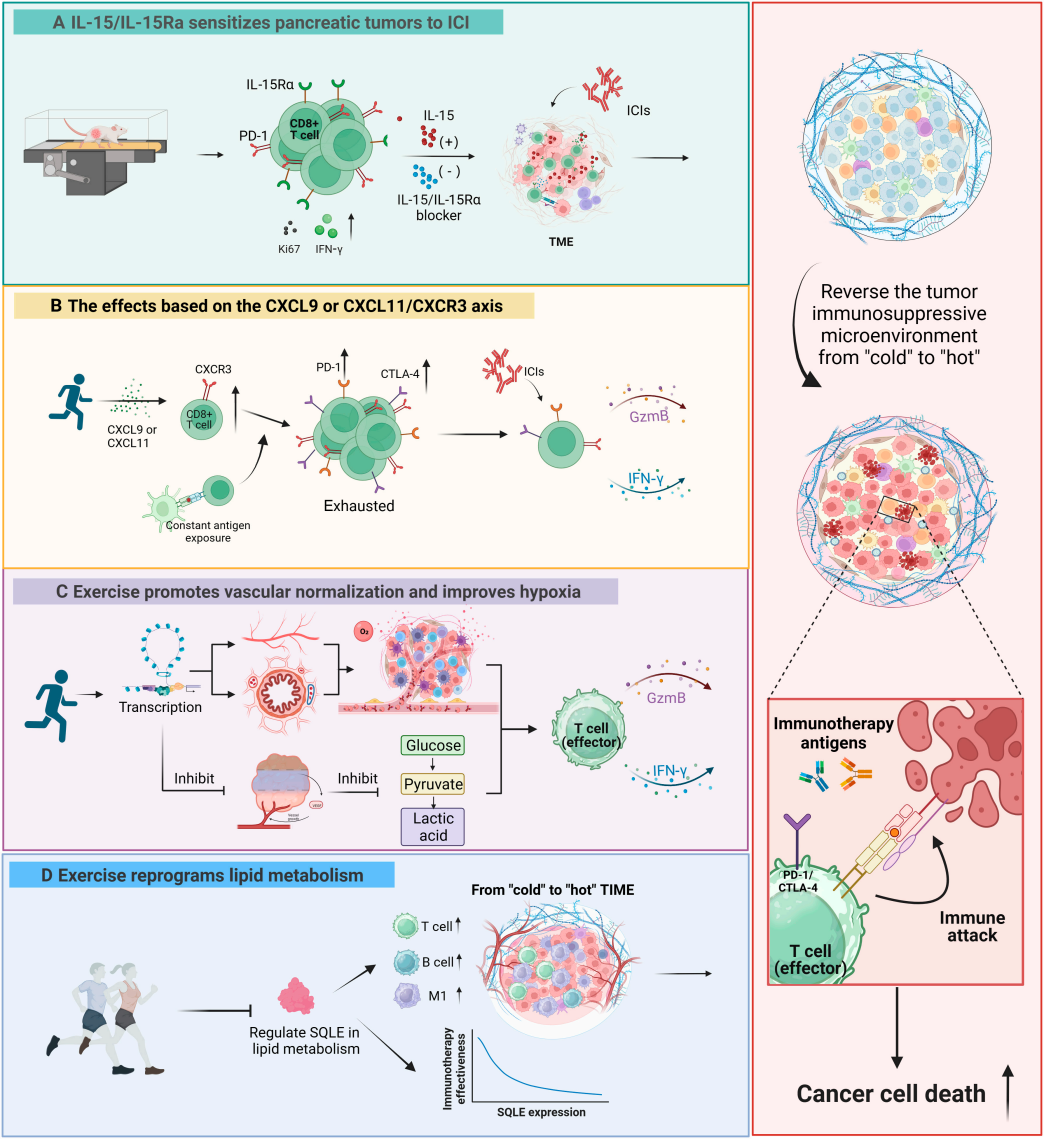
The mechanisms underlying enhanced immunotherapy efficacy through exercise. (A) The antitumor effects of exercise depend on IL-15 signaling pathway, thus enhancing the sensitivity of pancreatic tumor cells to immunotherapy. (B) Exercise increases the infiltration of CD8+ T cells through the CXCL9 or CXCL11/CXCR3 signaling pathway. (C) Exercise influences multiple transcriptional pathways, including promotion of vascular branching and normal vasoconstriction, which improves hypoxia in TME and enhances the efficiency of drug transport. Meanwhile, hypoxia-related transcription pathways are also inhibited, thereby reducing anaerobic glycolysis. (D) Exercise inhibited the expression of SQLE in lipid metabolism and reversed the TME. Simultaneously, a negative correlation was observed between the expression of SQLE and the immunotherapy efficacy. M1, M1 macrophage; TIME, tumor immune microenvironment; CTLA-4, cytotoxic T-lymphocyte-associated protein 4.

Exercise itself promotes the entry of CD8+ T cells into circulation, while certain cytokines, such as interleukins (ILs), mediate the enhanced functionality of these cells to optimize the systemic immune response [4]. Specifically, interleukin-15 (IL-15), acting as an upstream regulator, drives the proliferation and activation of IL-15Ra+ CD8+ T cells in pancreatic ductal adenocarcinoma [9]. Given the up-regulated PD-1 on these cells, aerobic exercise combined with PD-1 inhibitors reduced in situ/invasive pancreatic ductal adenocarcinoma weight by 20% to 30%. Blocking the IL-15/IL-15Rα axis led to a diminished synergistic effect (Fig. 1A). Intriguingly, the IL-15 super agonist mimicked these benefits, suggesting its therapeutic potential for future clinical applicability, especially for those unable to exercise [9].

Exercise also boosts immunotherapy by increasing chemokines like chemokine (C-X-C motif) ligand 9 (CXCL9) and 11 (CXCL11), as well as CD8+ T cells expressing chemokine (C-X-C motif) receptor 3 (CXCR3) which serves as the specific receptor for CXCL9 and CXCL11. Deletion of CXCR3 caused a considerable reduction of CD8+ T cells, highlighting the crucial role of the CXCL9 or CXCL11/CXCR3 axis in recruiting these cells to tumors [10]. Nevertheless, increased CD8+ T cells also led to more exhausted T cells that are characterized by PD-1 and cytotoxic T-lymphocyte-associated protein 4 expressions due to prolonged antigen exposure. Encouragingly, combining exercise with immune checkpoint blockade can reverse the TME and enhance effector CD8+ T cell infiltration and activity by increasing the granzyme B+ or interferon-γ+ (IFN-γ+) CD8+ T cells. This suggests that the reversal of TME from “cold” to “hot” improves the differentiation of CD8+ effector T cells and enhances the efficacy of immune checkpoint blockade (Fig. 1B). Notably, IFN-γ is considered a promotor of the expression of CXCL9 and CXCL11, suggesting a potential positive feedback loop [10].

### Normalizing vessels and ameliorating hypoxia

Abnormal angiogenesis results in the inefficient transport of drugs, while regular exercise normalizes vessels. Gene set enrichment analysis results in breast cancer patients and mice revealed that transcriptional pathways in correlation with vessel normalization were up-regulated in breast cancer patients conducting regular exercise, including branching involved in blood vessel morphogenesis and positive regulation of vasoconstriction. An extended portion of vessels associated with αSMA+ perivascular cells were also observed by means of immunofluorescence quantification [10]. The improved perfusion enabled a more efficient delivery of immunotherapy drugs. Additionally, the hypoxia-induced immunosuppressive TME limits the efficacy of immunotherapy, while aerobic exercise normalizes blood vessels, partly improving the insufficient oxygen supply. Apart from this, physical exercise could also alleviate hypoxia by down-regulating the expression of hypoxia-associated genes, hence shaping a more favorable TME with significantly higher levels of IFN-γ and granzyme B. Improved oxygen conditions reduced glycolysis and enhanced oxidative metabolism, which was beneficial to the function of effector T cells (Fig. 1C) [6].

### Reprogramming lipid metabolism

Exercise can enhance immunotherapy by modulating lipid metabolism. Squalene epoxidase (SQLE), as a rate-limiting enzyme in cholesterol biosynthesis, is associated with poor prognosis in non-small cell lung cancer patients due to its role in shaping the immune-cold phenotype. It is featured by a down-regulation of several immunomodulators (CXCL9/CXCL11, CXCR3, etc.) and a less infiltration of immune cells (CD4/CD8+ T cells, macrophages, dendritic cells, etc.). Given this, researchers further assumed that SQLE may be associated with responses to immunotherapy. In a combined public cohort, they found that SQLE was up-regulated in cases with poorer response, a finding also validated in their recruited non-small cell lung cancer cohort receiving ICIs. Intriguingly, all these negative effects were reversed by exercise for that it significantly inhibited SQLE expression, therefore heating the immune-cold TME and suppressing tumor growth (Fig. 1D) [11].

## Are We Ready for Customizing Exercise Prescriptions?

Despite the promising prospect, the synergistic effects between exercise and immunotherapy exhibit heterogeneity across diverse conditions, mainly due to the ambiguity of primary exercise parameters [12]. This further emphasizes the concept of “Exercise Prescription”, which means more refined and individualized exercise recommendations [13]. Future researches need to further investigate the impact of exercise on both local and systemic anticancer immune responses across diverse cancers. This will lay the foundation for subsequent exploration of optimal approaches combining different types, frequencies, and intensities of exercise with immunotherapy to achieve ideal efficacy. Moreover, we note that existing preclinical studies mostly performed exercise training in mice prior to receiving ICIs. This indicates that the impact of exercise at different stages (before, during, or after treatment) on the efficacy of immunotherapy also warrants attention from researchers.

Apart from those, the patient's pathological stage, physical condition, psychological state, acceptance of or preference for exercise are also factors worth considering in the prescription. Such insights will be instrumental in designing the most effective mode and dosage of exercise tailored to each individual patient to achieve optimal immunotherapy outcomes. We believe that it may be feasible to develop a cancer-patient-specific frequency–intensity–time–type principle based these factors [14]. In addition, considering the potential limitations of traditional resistance training for patients with poor physical condition, certain approaches like whole-body electromyostimulation training offer promising possibilities. By mimicking brain electrical signals to simulate muscle contractions, whole-body electromyostimulation is a joint-friendly and less-fatiguing form of exercise training, allowing for adjusting the frequency and intensity of stimulation to achieve thresholds [14].

As another key issue to consider, human prospective trials are crucial if we want to achieve clinical translation. Currently, the majority of research on exercise and its impact on immunotherapy efficacy are conducted on animal models, with limited clinical data available. Existing randomized controlled trials primarily focus on the physiological and psychological effects of exercise on cancer patients receiving immunotherapy [15–17], as well as the changes regarding immune and inflammatory biomarkers in blood samples [18,19]. However, overall survival, PFS, and ORR are supposed to be considered to assess the long-term influence of immunotherapy in combination with exercise training on cancer patients [20]. A clinical trial (NCT06298734) recruited 80 melanoma patients undergoing immunotherapy to investigate the effects of diet and exercise on efficacy and the microbiome. The intervention group received a high-fiber diet and weekly exercise, while the control group was not asked to change their diet or exercise habits. The primary outcome measure is feasibility and the secondary outcome measures are PFS, quality of life and ORR. No results have been disclosed yet. Another clinical trial named “Exercise to Boost Response to Checkpoint Blockade Immunotherapy” (NCT05358938) also considers “Impact of Exercise on Tumor Immunological Biomarkers” and “Pathological Complete Response (pCR)” as primary outcome measures. The research is still ongoing. Sustained efforts and investment will be essential to drive progress in this field.

## Concluding Remarks

By significantly altering the composition of immune cells and reversing the immune landscape, a synergistic combination of exercise and immunotherapy presents a promising therapeutic avenue for cancer treatment. Unfortunately, given the diversity of tumor types and patient characteristics, there is still a long way to go before applying this strategy in clinical practice.

Moreover, considering the multifaceted impacts of exercise on diverse physiological processes, the mechanisms underlying its synergy with immunotherapy extend far beyond the aforementioned pathways, deserving more profound explorations. In the future, as more valuable biological insights of the interaction between immunotherapy and exercise deepens, it is certain to pave the way for the discovery of novel diagnostic biomarkers or for the development of potent exercise-equivalent drugs, bringing about better quality of life and treatment outcomes for patients.
